# Correction to: Meaningful activities during COVID-19 lockdown and association with mental health in Belgian adults

**DOI:** 10.1186/s12889-021-11699-4

**Published:** 2021-09-15

**Authors:** Ellen Cruyt, Patricia De Vriendt, Miet De Letter, Peter Vlerick, Patrick Calders, Robby De Pauw, Kristine Oostra, Maria Rodriguez-Bailón, Arnaud Szmalec, Jose Antonio Merchán-Baeza, Ana Judit Fernández-Solano, Laura Vidaña-Moya, Dominique Van de Velde

**Affiliations:** 1 Faculty of Medicine and Health Sciences, Department of Rehabilitation Sciences, Occupational Therapy, Physiotherapy and Speech-language Pathology/Audiology, Ghent University, Corneel Heymanslaan 10, B3, entrance 46, 9000 Ghent, Belgium; 2 Department of Occupational Therapy, Artevelde University College, Ghent, Belgium; 3 Mental Health Research group, Frailty in Ageing Research Group, Vrije Universiteit Brussel, Brussels, Belgium; 4 Faculty of Psychology and Educational Sciences, Department of Work, Organization and Society, Ghent University, Ghent, Belgium; 5 Department of Physiotherapy (Occupational Therapy), University of Malaga, Málaga, Spain; 6 Psychological Sciences Research Institute, Université catholique de Louvain, Louvain-la-Neuve, Belgium; 7 Department of Experimental Psychology, Ghent University, Ghent, Belgium; 8 Research group on Methodology, Methods, Models and Outcomes of Health and Social Sciences (M3O), Faculty of Health Science and Welfare, University of Vic-Central University of Catalonia (UVIC-UCC), 08500 Vic, Spain; 9 Department of Occupational Therapy. School of Health Sciences, Catholic University of Murcia, Murcia, Spain; 10 Research Group GrEUIT, Escola Universitària d’Infermeria i Teràpia Ocupacional de Terrassa (EUIT), Universitat Autònoma de Barcelona, Terrassa, Spain

**Correction to: BMC Public Health 21, 622 (2021)**

**10.1186/s12889-021-10673-4**

It was highlighted that in the original article [1] the given name and family names of the authors were interchanged. The original article has been updated.
